# In Vitro and in Vivo Characteristics of Fluorapatite-Forming Calcium Phosphate Cements

**DOI:** 10.6028/jres.115.020

**Published:** 2010-08-01

**Authors:** Shozo Takagi, Stan Frukhtbeyn, Laurence C. Chow, Akiyoshi Sugawara, Kenji Fujikawa, Hidehiro Ogata, Makoto Hayashi, Binnai Ogiso

**Affiliations:** American Dental Association Foundation, Paffenbarger Research Center, National Institute of Standards and Technology, Gaithersburg, MD 20899, U.S.A; Nihon University School of Dentistry, and Sugawara Dental Clinic, Tokyo, Japan; Nihon University School of Dentistry, and Fujikawa Dental Office, Tokyo, Japan; Nihon University School of Dentistry, Tokyo, Japan

**Keywords:** animal study, biocompatible, fluorapatite-forming calcium phosphate cements, high crystallinity, non-resorbable

## Abstract

This study reports for the first time *in vitro* and *in vivo* properties of fluorapatite (FA)-forming calcium phosphate cements (CPCs). The experimental cements contained from (0 to 3.1) mass % of F, corresponding to presence of FA at levels of approximately (0 to 87) mass %. The crystallinity of the apatitic cement product increased greatly with the FA content. When implanted subcutaneously in rats, the *in vivo* resorption rate decreased significantly with increasing FA content. The cement with the highest FA content was not resorbed in soft tissue, making it the first known biocompatible and bioinert CPC. These bioinert CPCs might be useful for applications where slow or no resorption of the implant is required to achieve the desired clinical outcome.

## 1. Introduction

Since their discovery two and half decades ago [1], calcium phosphate cements (CPCs) have been the subject of considerable interests in the field of bone graft biomaterials. CPC materials have been universally found to be biocompatible and osteoconductive [2–7]. This leaves CPC’s bio-resorption characteristics as one of the remaining important properties to be more fully understood and controlled in order to achieve optimum CPC-to-bone conversion. CPCs of different compositions can form different end products such as hydroxyapatite (HA), octacalcium phosphate, and dicalcium phosphate dihydrate (DCPD), also known as brushite. An important *in vivo* property of HA-forming CPCs is that the HA formed does not dissolve spontaneously in a normal physiological fluid environment, yet is resorbable under cell-mediated acidic conditions. Although DCPD is soluble in normal physiological fluids, studies have shown that resorption of DCPD-forming CPC was also essentially cell-mediated, mainly due to conversion of the DCPD to an apatitic phase *in situ*. CPCs with different resorption rates may be especially suitable for different clinical applications.

For some clinical applications, specifically endodontic applications, such as root end fill, perforation repair, etc., it is desirable to have CPCs that are biocompatible and osteoconductive, yet non-bioresorbable in soft and hard tissues. Since *in vivo* resorption is a result of dissolution in a cell-mediated acidic environment, CPCs that form products that have little or practically no solubility in such acidic conditions can be expected to be essentially non-resorbable. It is well described in the literature that fully or partially fluoridated hydroxyapatite materials have significantly lower solubility in acids [8]. Thus, fluorapatite (FA)-forming CPCs can be expected to have much lower resorbability than HA-forming CPCs. In this study, we report for first time, development of FA-forming CPCs. Their physicochemical properties as well as preliminary in *in vivo* resorption characteristics are described.

## 2. Materials and Methods

All acronyms used in study 1 and study 2 are shown in Table 1.

### 2.1 Cement Powder and Liquid

CPC powder used in the study was a conventional CPC [9] consisting of equimolar amounts of tetracalcium phosphate (TTCP), Ca_4_(PO_4_)_2_O, and dicalcium phosphate anhydrous (DCPA), CaHPO_4_. TTCP was prepared by heating an equimolar mixture of commercially obtained DCPA (Baker Analytical Reagents, J. T. Baker Chemical Co., Phillipsburg, NJ) and CaCO_3_ (J. T. Baker Chemical Co.)^1^ at 1500 ºC for 6 h in a furnace and quenched at room temperature. All chemicals were reagent grade and used as received. The TTCP and DCPA powders were ground individually in a planetary ball mill (PM4, Brinkman, NY). The TTCP was ground dry and DCPA was ground in 95 % ethanol for 24 h to obtain the desired median particle sizes. The particle sizes of TTCP and DCPA were measured using a centrifugal particle size analyzer (SA-CP3, Shimadzu, Kyoto, Japan) with an estimated standard uncertainty of 0.2 μm. The median particle sizes of TTCP and DCPA used in this study were 17 μm and 1 μm, respectively.

Cement liquid was a 2 mol/L H_3_PO_4_ solution containing either 1 mol/L (L1), 4 mol/L (L2) or 8 mol/L (L3) hydrogen fluoride (HF) (B&A, Industrial Chemical Division, Morristown, NJ).

Three experimental FA-forming CPCs, FA-CPC1, FA-CPC2 and FA-CPC3 were prepared by mixing the CPC powder with the L1, L2 or L3 liquid at a powder-to-liquid ratio (P/L) of 2. At this P/L ratio, the amount of F supplied by the cement liquid would account for approximately (0.5, 2, and 4) mass % of the total solid mass, compared to the theoretical F content of pure FA of 3.8 mass %. Control CPC (CPC0) was prepared by mixing the CPC powder with the 2 mol/L H_3_PO_4_ solution without HF at P/L = 2.

### 2.2 Study 1—*In Vitro* Study of FA-CPCs

The three experimental FA-forming CPCs, FA-CPC1, FA-CPC2 and FA-CPC3 and the control CPC, CPC0, were prepared by mixing the respective powder and liquid on a Teflon slab for 20 s. After mixing the pastes were placed in stainless steel molds (6 mm D, 3 mm H) for 1 h. Hardened FA-CPCs were removed from molds and immersed for 23 h in a physiological-like solution (PLS) containing 1.15 mmol/L Ca, 1.2 mmol/L P, 133 mmol/L NaCl, 50 mmol/L HEPES, and pH adjusted to 7.4. The fluoride (F), calcium (Ca), and phosphate (P) contents of the 24 h FA-CPCs were determined by dissolving the sample in 0.5 mol/L HClO_4_ and the acid solution was analyzed for F using a specific ion electrode, and Ca and P using spectrophotometric methods [10]. The phases present in the 24 h FA-CPCs were characterized by powder x-ray diffraction (XRD). The XRD patterns were recorded (Rigaku DMAX 2200, Danvers, MA, U.S.A.) using graphite-monochromatized CuKα radiation (λ = 0.154 nm) generated at 40 kV and 40 mA. The specimen was scanned from (20 ° to 40 °) 2θ in a continuous mode (2° 2θ min^−1^, time constant 2 s). The surface morphology was observed using scanning electron microscopy (SEM) (JEOL JSM-5300, JOEL U.S.A., Inc., Peabody, MA) under the condition of 15 kV and 58 mA.

In this study, the standard deviation is considered as the standard uncertainty for all experimentally measured values.

In a separate experiment, fully cured FA-CPC3 (formulation with the highest F content) samples were analyzed using the KOH extraction method of Caslavska [11] for the amounts of F present in the sample in two possible forms: (1) F incorporated into the apatite crystal structure and (2) F present in the form of calcium fluoride.

### 2.3 Study 2—Preliminary *In Vivo* Resorption Study Using a Rat Model [12]

**Fig. 1:**
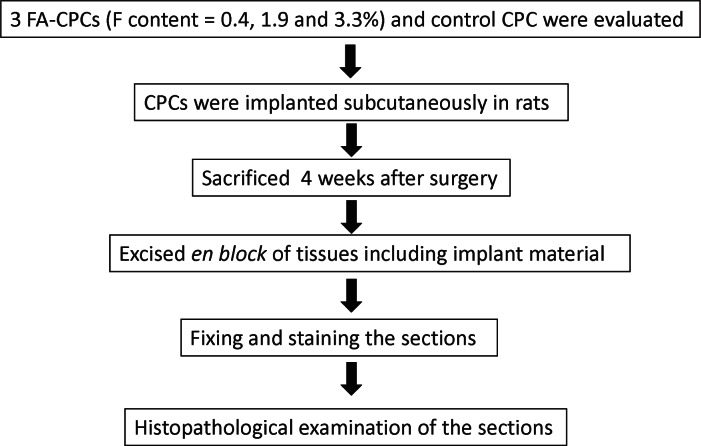
Experimental procedure of the animal study.

**Fig. 2:**
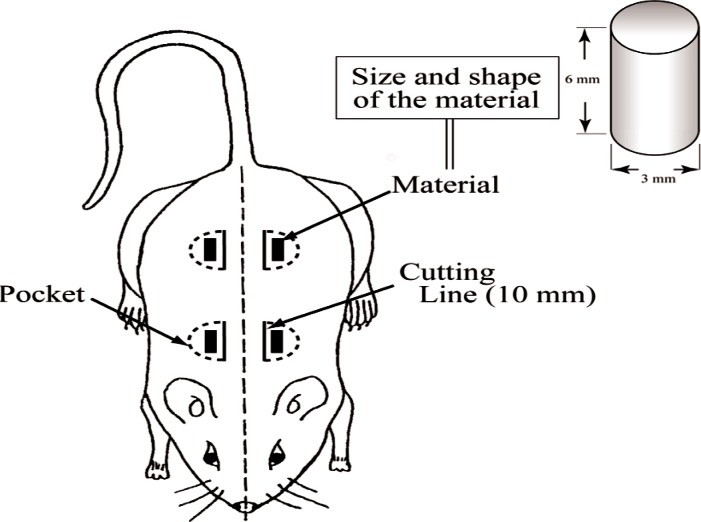
Schematic drawing of the pocket created in back area of rat.

**Fig. 3:**
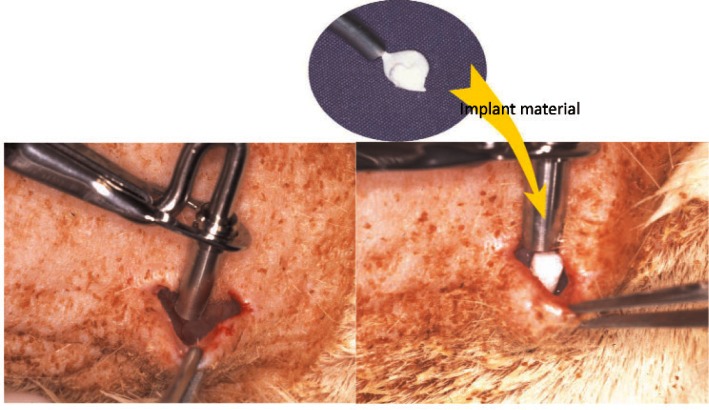
Grafting procedure of the experiment.

The animal experiments were conducted, with approval of the Animal Experimentation Committee at Nihon University School of Dentistry, in the animal and cell culture laboratories at Nihon University School of Dentistry. The study fully complied with the “Guidelines for Animal Experimentation Committee at Nihon University School of Dentistry” and with the NIH’s “Standards for Humane Care and Use of Laboratory Animals by Foreign Institutions.” Experimental protocols are shown in Fig. 1. Each experimental FA-CPC material was tested in five adult Donryu rats with an average body weight of 200 g to 250 g. All experimental procedures on given animals were completed under aseptic conditions. Each animal was anesthetized with a pentobarbital sodium injection at a dose of 1.5 mg/kg body weight. Under the general anesthesia, the back area of the rat was shaved and swabbed with 70 % volume fraction ethanol. Four subcutaneous pockets were created, two on each side of the backbone, to implant the experimental CPCs. Four horizontal incisions approximately 15 mm in length were made along each side of the back bone, and subcutaneous skin pockets were created by blunt dissection (Fig. 2). The pockets were separated by 40 mm to 50 mm. Each cylindrical shaped sample (3 mm diameter and 6 mm length) was inserted into a pocket of subcutaneous tissues as shown in Fig. 3, and then the pocket was closed with interrupted sutures. Four weeks after surgery, the animals were sacrificed and the tissues including the test materials were excised *en block*. Tissues were fixed in 10 % neutralized-buffered formalin, decalcified with 10 % formic acid for 14 d and embedded in paraffin. This decalcification period for the sample was longer than those of normal conditions. Subsequently, paraffin embedded blocks of decalcified specimens were cut into 3 μm to 4 μm cross-sections, and stained with hematoxylin and eosin.

Histopathological features of each specimen were observed using an optical microscope.

## 3. Results

### 3.1 Study 1

The KOH extraction experiment [11] revealed negligible amounts of KOH extractable F in the FA-CPC3 samples, indicating that all of the F was incorporated into the apatite structure. The measured F contents in the three FA-forming CPCs were (0.4, 1.8, and 3.1) mass % (Table 1), which are close to the expected values of (0.5, 2, and 4) mass %, respectively. The molar Ca/P ratio of the samples, which ranged from (1.37 to 1.69), increased with increasing F content (Table 2).

**Fig. 4:**
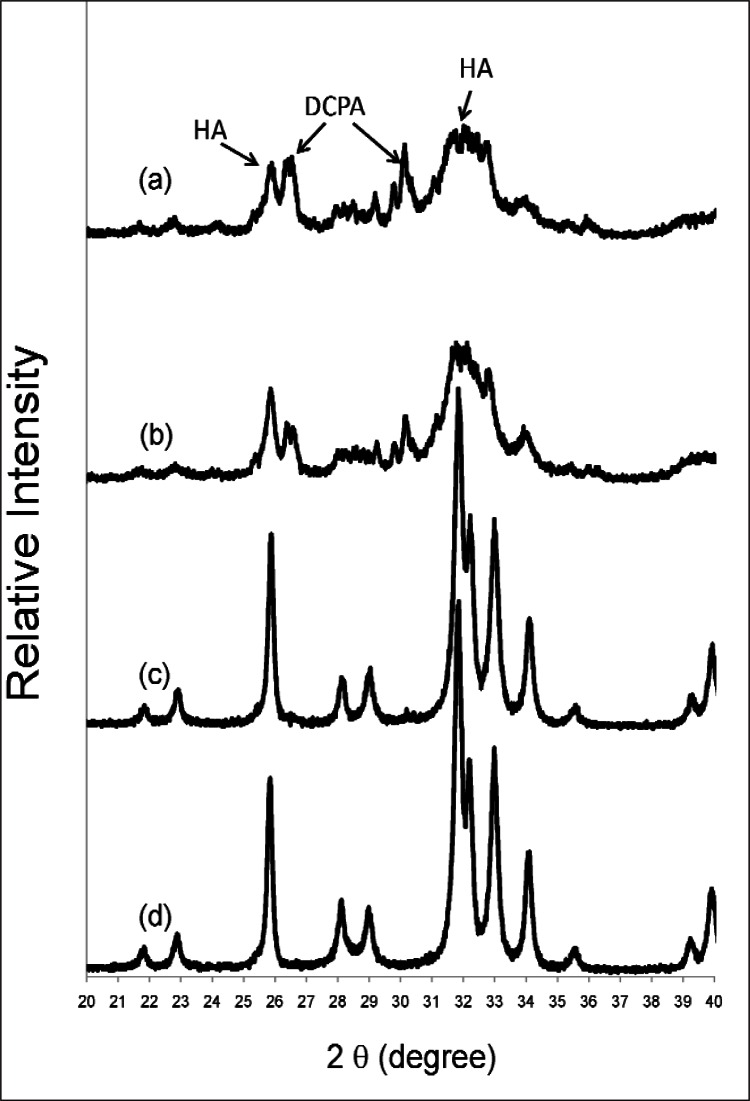
Powder x-ray diffraction patterns of CPC0 and FA-CPCs: (a) CPC0, (b) FA-CPC1, (c) FA-CPC2 and (d) FA-CPC3.

The powder XRD pattern of the control, CPC0, showed that the sample contained poorly crystalline HA and unreacted DCPA (peaks at 30.19 °, 26.59 °, and 26.43 ° 2θ) (Fig. 4). The presence of the latter can be attributed by the use of the highly acidic and phosphate-rich cement liquid (2 mol/L H_3_PO_4_), which preferentially consumed TTCP, leaving some DCPA unreacted. The powder XRD pattern of FA-CPC1 is similar to that of CPC0, but with somewhat better crystalline HA and less unreacted DCPA. In contrast, FA-CPC2 and FA-CPC3 showed highly crystalline apatitic materials as the only phase present. The XRD patterns show that the crystallinity increased with increasing HF concentration of the cement liquid.

**Fig. 5:**
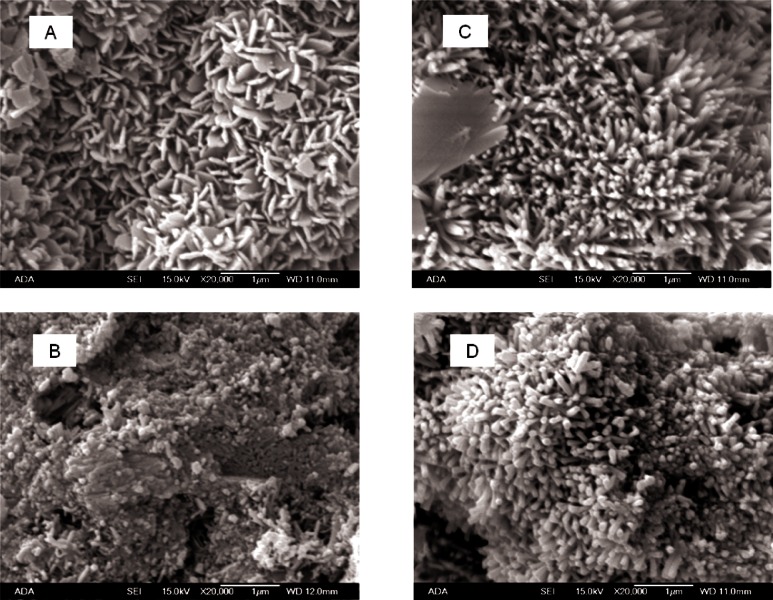
Surface morphology of CPC0 and FA-CPC3 specimens: (A) flat surface of CPC0, (B) fractured surface of CPC0, (C) flat surface of FA-CPC3 and (D) fractured surface of FA-CPC3.

After 23 h incubation in PLS, both the FA-CPCs and control CPC samples were covered with plate and rodshaped crystals (Figs 5a and 5c), similar to those reported in previous studies [13, 14]. Fractured surfaces of the control CPC0 showed small and less well-formed crystals, while FA-CPC3 showed mostly larger rod-like crystals. The F contents of the four CPC materials (Table 1) were (0, 0.4, 1.8 and 3.1) mass %. These correspond to the presence of FA in the cement products at levels of approximately (0, 10, 50, and 87) mass %. Results from the present study indicate that the F effects on crystallinity started to become highly pronounced somewhere between FA-CPC1 (10% FA) and FA-CPC2 (50 % FA). Further studies should be designed to determine the minimum FA content needed to produce significant effects.

### 3.2 Study 2

**Fig. 6:**
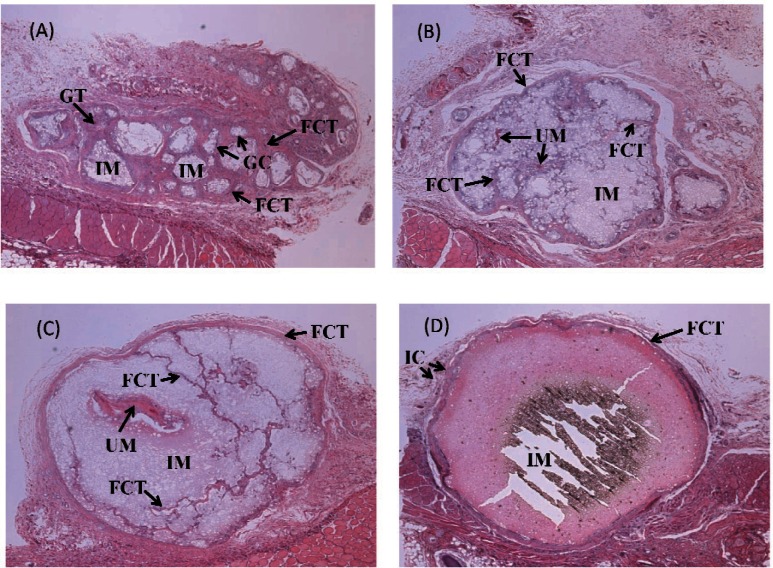
Histopathological features of CPC0 and FA-CPCs. (A) CPC0, (B) FA-CPC1, (C) FA-CPC2, and (D) FA-CPC3. Implanted material (IM), Fibrous Connective Tissue (FCT), Giant Cell (GC), Granuation Cell (GT), Undecalcified Material (UM), and Infiltrated Cell (IC).

All FA-CPC implants, but not the control CPC0, retained the original cylindrical shape and were encapsulated by thin fibrous connective tissues (FCT) with small numbers of infiltrated cells (IC) (Fig. 6). Significant differences in tissue response to the four types of implants were noted as described below.

#### 3.2.1 CPC0 Samples (Fig. 6A)

Significant resorption of CPC0 had occurred, and the implanted material (IM) was well separated into small domains which were covered by FCT. The remaining IM was completely decalcified by the 14-d decalcification period in 10 % formic acid conducted in the specimen preparation process. Small numbers of macrophage and foreign body giant cells (GC) were found adjacent to the material. Granulation tissues (GT) were also formed in the implant area. The tissue reaction of the material was mild.

#### 3.2.2 FA-CPC1 Samples (Fig. 6B)

FA-CPC1 resorbed considerably less than CPC0, but FCT was clearly formed throughout the implant area. Although the histopathological reactions of the surrounding tissues to FA-CPC1 were basically similar to the reaction to other FA-containing CPCs, inflammatory reaction was relatively few or negligible. The FCT surrounding the FA-CPC1 was very thin in comparison to the FCT formed over other experimental materials. Most of the IM was decalcified, but small areas of the undecalcified material (UM) can be seen. The tissue reaction was extremely mild.

#### 3.2.3 FA-CPC2 Samples (Fig. 6C)

The implanted FA-CPC2, which consisted of approximately 50 % FA, was surrounded by relatively thin and dense FCT. Slight resorption of the implanted material had occurred, resulting in FCT formation in highly confined spaces within some areas of the IM. Less amount of FCT was formed compared to that observed in the FA-CPC1 group. Very few inflammatory cells were found adjacent to the material. Some UM was seen in the implant area. The tissue reaction of the material was very mild.

#### 3.2.4 FA-CPC3 Samples (Fig. 6D)

The implanted FA-CPC3, which has the composition of nearly pure FA, was not resorbed. The filling area was surrounded by relatively thick and dense FCT with small numbers of inflammatory cells. No FCT or other tissues were found within the implanted material. The implanted material remained completely un-dissolved by the decalcification step in the sample preparation, most likely due to the low acid solubility of FA. The tissue reaction of the material was gentle.

In general, all of the experimental FA-CPCs demonstrated good biocompatibility and shape-integrity when implanted in subcutaneous tissues.

## 4. Discussion

### 4.1 Study 1

An equimolar mixture of TTCP and DCPA was used as the solid component of all of the CPC materials investigated in the present study. The control CPC (CPC0), prepared by mixing the TTCP+DCPA powder with a F-free 2 mol/L H_3_PO_4_ solution, formed a poorly crystalline apatitic product together with some unreacted DCPA (Fig. 4a). This result is consistent with literature reports [15, 16] that Ca-deficient hydroxyapatite was the main reaction product from cements with this same TTCP+DCPA mixture as the solid component and water or a sodium phosphate solution (pH from 2.5 to 9) as the cement liquid. The presence of unreacted DCPA in the present study was probably due to the high acidity of the cement liquid (2 mol/L H_3_PO_4_), leading to exhaustion of TTCP, the more alkaline component in the cement powder, before all DCPA could be reacted. Inclusion of HF in the cement liquid produced two significant effects on the cement products. Firstly, the amount of unreacted DCPA decreased with increasing HF concentration. No unreacted DCPA was found in FA-CPC2 and FA-CPC3 (Figs. 4c and 4d). Secondly, the crystallinity of the apatitic product increased greatly with increasing HF concentration. The powder XRD patterns (Fig. 4) showed that both FA-CPC2 and FA-CPC3 are highly crystalline apatitic materials, whereas CPC0 was a poorly crystalline apatite. This latter observation is in good agreement with numerous previous findings [17–20] that F, when incorporated into the apatite crystal structure, greatly increased the crystallinity.

The present study is the first report of CPCs that form FA as a significant phase in the product. In this initial study, HF was used as the F source to minimize the number of components in the cement system. However, this leads to high acidity of the cement paste during setting. Results from ongoing studies showed that NaF can also be used as a source of F. Further, FA-forming CPCs can be formulated from several different calcium phosphates powder mixtures that would have acidic, neutral, or alkaline properties during and after setting. The FA-CPCs may find clinical applications where slow or no *in vivo* resorption is desired.

### 4.2 Study 2

Because of the high acidity and high initial HF concentration in the cement liquid, one may expect that implantation of the experimental FA-CPC pastes in subcutaneous tissues, would incite negative tissue reactions. The results obtained from the study showed that the histopathological reactions of FA-CPCs were nearly identical and similar to the CPC0 that did not contain HF. These results were also similar to those of the conventional TTCP+DCPA cement that used water as the cement liquid [4,12] or to sintered hydroxyapatite [2,4]. In all cases, the implanted CPC material was surrounded by FCT with either negligible or small numbers of inflammatory cells. All of the materials used in this study showed high biocompatibility.

Histopathological examination results showed a clear trend of *in vivo* resorption for the four experimental CPCs as follows. On the one end, the F-free CPC0 showed significant resorption, and the implanted material was well separated into small domains that were covered by FCT. On the other end, the implanted FA-CPC3, which had the composition of nearly pure FA, was not resorbed. No FCT or other tissues were found within the implanted material. The *in vivo* properties of the other 2 FA-CPCs fell in between the two extremes. The lack of resorption of FA-CPC3 is likely to be the result of its insolubility in the acidic environment produced by inflammatory or other giant cells. This material appears to be the first calcium phosphate cement that is chemo-mechanically stable in soft tissues, making it a biocompatible and bioinert material. Further studies are needed to determine whether FA-CPC3 and possibly other FA-forming CPCs also exhibit similar *in vivo* properties when implanted in bone. These bioinert CPCs might be useful for applications where slow or no resorption of the implant is required to achieve desired clinical outcome.

## 5. Conclusion

Previous studies have demonstrated that CPC materials were biocompatible and osteoconductive. The results obtained from the present study showed that FA-CPCs are highly biocompatible with subcutaneous tissues. The resorption rate of FA-CPCs appears to decrease with increasing FA content, and suggests that the FA-CPC3, which has a composition of nearly pure FA, is non-resorbable *in vivo*.

## Figures and Tables

**Table 1:** Acronyms used in Study 1 and Study 2

Calcium phosphate cement	CPC	Granulation tissue	GT
Dicalcium phosphate anhydrous	DCPA	Hydroxyapatite	HA
Dicalcium phosphate dihydrate	DCPD	Infiltrated cell	IC
Fluorapatite	FA	Implanted material	IM
Fibrous connective tissue	FCT	Tetracalcium phosphate	TTCP
Giant cell	GC	Undecalcified material	UM

**Table 2:** Fluoride content and Ca/P ratio

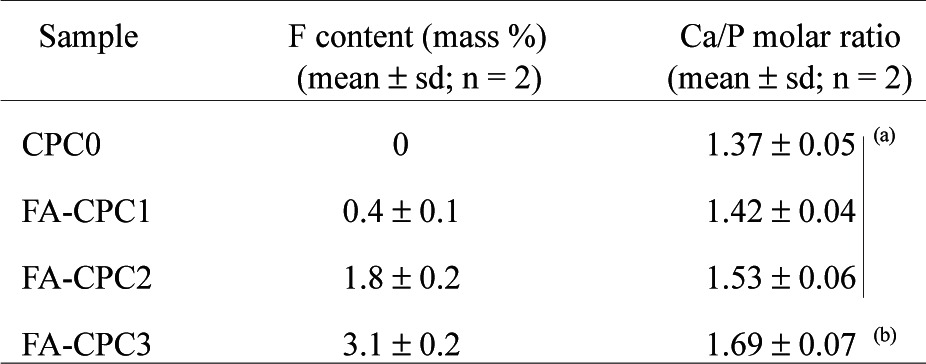

(a) Values connected by a line are not significantly (p > 0.05) different.

(b) Significantly different (p < 0.05) from other Ca/P values.

## Footnotes

1 Certain commercial materials and equipment are identified in this paper to specify the experimental procedure. In no instance does such identification imply recommendation or endorsement by the National Institutes of Health or American Dental Association Foundation or National Institute of Standards and Technology or Nihon University or that the material or equipment identified is necessarily the best available for the purpose.
